# Metastatic spinal cord compression (MSCC) treated with palliative decompression: Surgical timing and survival rate

**DOI:** 10.1371/journal.pone.0190342

**Published:** 2017-12-29

**Authors:** Wan-Yu Lo, Shu-Hua Yang

**Affiliations:** 1 Department of Orthopedics, National Taiwan University Hospital, Taipei, Taiwan; 2 Institute of Toxicology, College of Medicine, National Taiwan University, Taipei, Taiwan; George Washington University, UNITED STATES

## Abstract

**Background:**

Metastatic spinal cord compression (MSCC) treatment depends on life expectancies. Data regarding palliative decompression outcomes is scarce. We demonstrate that surgical timing has a significant impact on survival in MSCC patients treated with palliative decompression.

**Methods:**

Eighty-nine consecutive MSCC patients at a tertiary referral medical center were enrolled between January 2012 and February 2016. Wide laminectomy was performed for tumors invading the vertebral body. Debulking surgery was done for tumors damaging the posterior column of the spine. Patient records were retrospectively analyzed.

**Results:**

Better survival was observed in patients with preoperative intact motor function (Group A, n = 37) than in those with motor deficit (Group B, n = 52, *p* = 0.0031). In Group B, survival was better in those who underwent surgery within 7 days of motor deficit onset than in those who underwent surgery 7 days after onset (*p* = 0.0444) and in postoperative ambulant patients than in nonambulant patients (*p* = 0.0120). In Group B, Frankel grade improved in patients who underwent surgery within 48 h than in those who underwent surgery after 48 h (*p* = 0.0992). Group A patients had a shorter hospital stay and higher revised Tokuhashi score than Group B patients. Overall survival was better in patients with a lower Tomita score (≤5, *p* = 0.0012), higher revised Tokuhashi score (≥9, *p* = 0.0009), better preoperative Frankel grade (*p* < 0.0001), and younger age (≤55 years, *p* = 0.0179). There were no significant differences in age, sex, tumor type, involved vertebrae level, Tomita score, intraoperative blood loss, operation time, incidence of infection, and postoperative complications between groups.

**Conclusion:**

We can improve the survival of MSCC patients with palliative decompression before motor deficits occur. After motor deficit onset, survival can still be improved with surgery within 7 days. Overall survival was better in patients aged ≤55 years.

## Introduction

Metastatic spinal cord compression (MSCC) is a challenging condition for surgeons to treat. It develops in 5%–10% of all cancer patients and in 40% of patients with preexisting nonspinal bone metastasis [1].

Treatments for patients with MSCC differ based on their life expectancies [2, 3]. To improve the quality of life, patients with a life expectancy of more than 3–6 months may undergo surgery [4, 5]. The increasing number of patients with a longer life expectancy has raised the demand for decompression surgery.

Factors influencing surgical outcomes are of utmost importance. Previous studies demonstrated that surgery within 48 h of the onset of motor deficits led to better neurological outcomes [6, 7, 8]. In addition, palliative decompression followed by radiotherapy led to better survival and functional outcome than radiotherapy alone [4, 8, 9]. A lower preoperative Tomita score and a higher revised Tokuhashi score also led to better prognosis [10, 11, 12]. There is a strong correlation between the preoperative ambulatory function and survival rate. The better the preoperative ambulatory function the higher the survival rate [13, 14, 15, 16]. Studies also agree that a better postoperative ambulatory function leads to a higher survival rate [11, 17].

However, there are limited data on the effects of surgical timing on patient survival or the outcomes of palliative decompression. Studies discussing surgical timing have focused on neurological recovery instead of survival. Studies discussing survival did not mention surgical timing.

The current retrospective cohort study including 89 consecutive MSCC patients who underwent palliative decompression between January 2012 and February 2016 at a tertiary referral medical center. The outcomes were evaluated by survival time and Frankel grade [18] improvement. This study demonstrates that surgical timing can have a significant impact on the survival rate of MSCC patients treated with palliative decompression. Other factors, which may influence patient survival, are discussed, including age, postoperative Frankel grade [18], the Tomita score [3], and the revised Tokuhashi score [12, 19].

## Materials and methods

### Inclusion and exclusion criteria

The protocol and the request for the waiver of informed consent have been approved by the Research Ethics Committee D of the National Taiwan University Hospital, and were fully ratified in the 55th meeting of Research Ethics Committee. The committee is organized under, and operates in accordance with, the Good Clinical Practice guidelines and governmental laws and regulations. The data were analyzed anonymously.

Consecutive patients with MSCC, who were diagnosed by radiographic findings and clinical symptoms, underwent palliative decompression between January 2012 and February 2016 at a tertiary referral medical center. The patients were followed for at least 2 years after palliative decompression, and most of them died within 2 years. The inclusion criteria were MSCC with a known or unknown primary cancer treated with palliative decompression and a tissue-proven diagnosis of a solid primary tumor or cancers of the blood and lymph glands. The exclusion criteria included patients with spinal metastasis who received corpectomy for more aggressive removal of metastatic tumor, vertebroplasty or kyphoplasty procedures without decompression, or only radiotherapy. Patients with central nervous system origin of metastatic tumors were also excluded.

### Surgical techniques

All patients underwent palliative decompression and posterior stabilization. For tumors invading the vertebral body, wide laminectomy was performed; for tumors destroying the posterior column of the spine, debulking surgery was performed. Patients received postoperative adjuvant radiotherapy, as required. The surgical indications were deficit in sensory, motor, or sphincter function, as well as back pain caused by instability.

### Clinical evaluations

All data were retrospectively collected through medical records including age, sex, survival time, Frankel grade change (Table 1) [18], type of primary tumor, location of the metastatic tumor involving the vertebrae, length of hospital stay, blood loss, operation time, complications, Tomita scores (Table 2) [3, 10], and revised Tokuhashi scores (Table 3) [12, 19]. The type of primary tumor was classified into three groups—rapid growth tumors (lung cancer, gastric cancer, esophageal cancer, cancer of the ampulla of Vater, cholangiocarcinoma, and hepatocellular carcinoma), moderate growth tumors (myeloma, leukemia, lymphoma, oral cancer, renal cell carcinoma, and others not listed), and slow growth tumors (colorectal cancer, breast cancer, and prostate cancer), which were determined using the revised Tokuhashi score system [12, 19]. The location of the metastatic spinal tumor was divided into cervical, upper thoracic (T1–T6), lower thoracic (T7–T12), lumbar, and sacral. When more than one location was involved, the most proximal location operated was representative. The length of hospital stay had acute and chronic stage, i.e., postoperative hospitalization in an orthopedic or neurosurgical ward and total hospitalization (including referral to oncological and/or internal medicine ward), respectively.

**Table 1:** Frankel grade classification.

Frankel grade	Motor	Sensory
A	Complete loss	Complete loss
B	Complete loss	Preserved
C	Incomplete motor function (non-ambulatory)	Preserved
D	Fair to good motor function (ambulatory)	Preserved
E	Normal	Normal

**Table 2:** Tomita scoring system.

Point	Primary tumor	Visceral metastases*	Bone metastases**
1	Slow growth	–	Solitary or isolated
2	Moderate growth	Treatable	Multiple
4	Rapid growth	untreatable	–

*No visceral metastases = 0 point.

**Bone metastases includes spinal metastases

**Table 3:** Revised Tokuhashi scoring system.

Characteristics	Score
**1. General condition (performance status)**	
Poor (PS 10–40%)	0
Moderate (PS 50–70%)	1
Good (PS 80–100%)	2
**2. Number of extraspinal bone metastases foci**	
≥3	0
1–2	1
0	2
**3. Number of metastases in the vertebral body**	
≥3	0
2	1
1	2
**4. Metastases to major internal organs**	
Unremovable	0
Removable	1
No metastases	2
**5. Primary cancer site**	
Lung, osteosarcoma, stomach, bladder, esophagus, pancreas	0
Liver, gallbladder, unidentified	1
Others	2
Kidney, uterus	3
Rectum	4
Thyroid, breast, prostate, carcinoid tumor	5
**6. Palsy**	
Complete (Frankel A, B)	0
Incomplete (Frankel C, D)	1
None (Frankel E)	2

Criteria of predicted prognosis: total score (TS) 0–8 = <6 months, TS 9–11 = ≥6 months, TS 12–15 = ≥1 year.

### Statistical analysis

Statistical analyses were performed using MedCalc for Windows version 16.8 (MedCalc Software, Ostend, Belgium). Survival data were computed using Kaplan–Meier survival analysis. All data that rejected normality were analyzed using Mann–Whitney U test, whereas those with accepted normality were analyzed using an independent *t* test. Categorical data were analyzed using Fisher’s exact test or Chi-square test. Paired data not normally distributed were analyzed using Wilcoxon signed-rank test. All *p* values of <0.05 were considered statistically significant.

## Results

**Fig 1:**
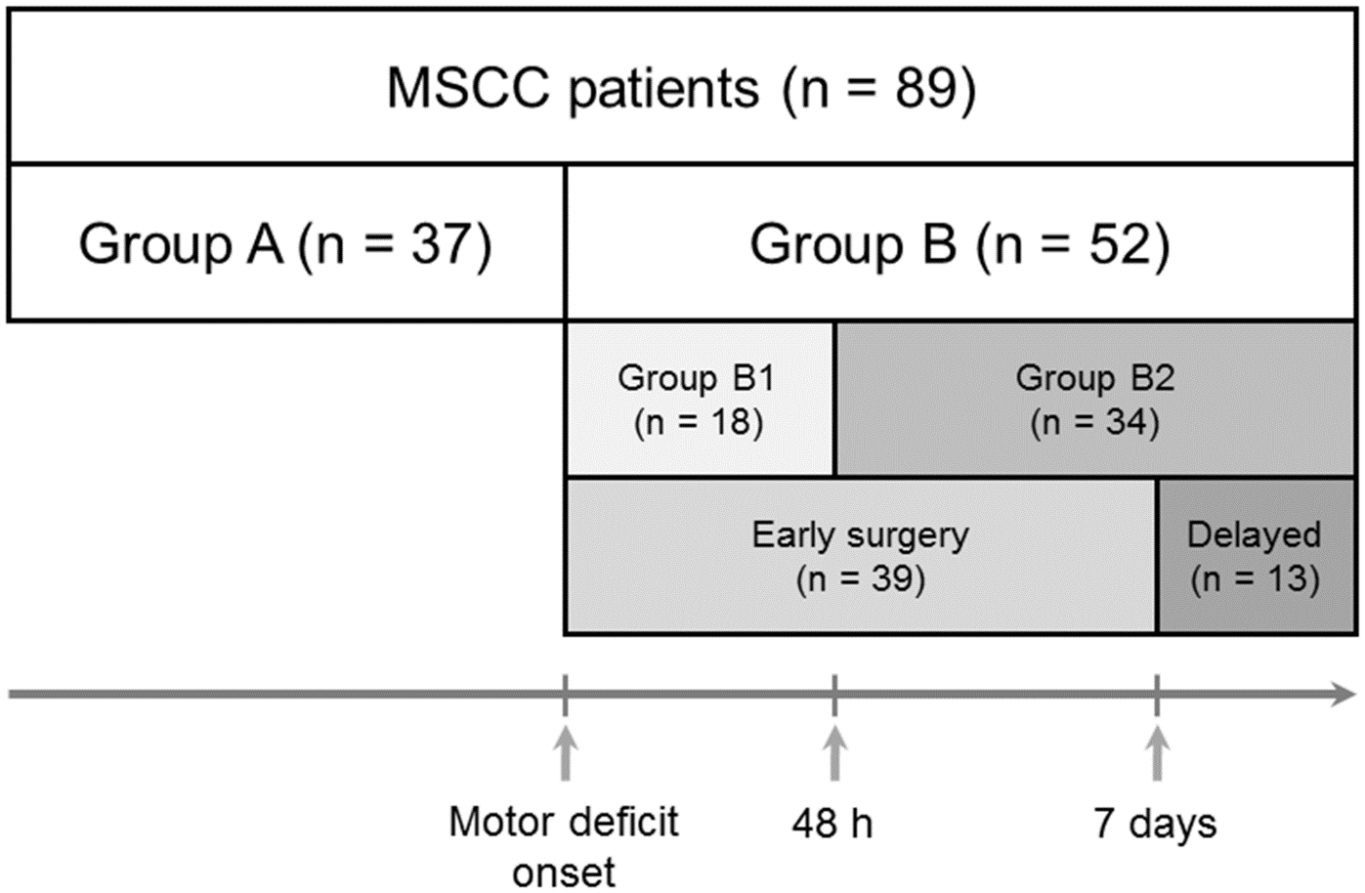
Classification of patients. Patients were divided into a preoperative intact motor function group (Group A, n = 37) and a preoperative motor deficit group (Group B, n = 52). Group B was further subdivided into a group that underwent surgery within 48 h of onset of motor deficit (Group B1, n = 18) and one that underwent surgery after 48 h (Group B2, n = 34). Another categorization of Group B was based on the operation performed within or after 7 days (early surgery group, n = 39; delayed surgery group, n = 13).

Eighty-nine (89) patients who underwent palliative decompression for MSCC between January 2012 and February 2016 were enrolled, including 35 (39.3%) females and 54 (60.7%) males. Mean age at the time of surgery was 56.8 ± 1.3 years (range 27–85). Bone metastasis was confirmed with histology and magnetic resonance imaging. Patients were divided into a preoperative intact motor function group (Group A, n = 37) and a preoperative motor deficit group (Group B, n = 52). Group B was further subdivided into a group that underwent surgery within 48 h of onset of motor deficit (Group B1, n = 18) and one that underwent surgery after 48 h (Group B2, n = 34). Another categorization of Group B was based on the operation performed within or after 7 days (early surgery group, n = 39; delayed surgery group, n = 13; Fig 1).

### Patient characteristics

There were no statistically significant differences between Groups A and B in age (*p* = 0.54), sex (*p* = 1.00), preoperative Tomita score (*p* = 0.0992), blood loss (*p* = 0.19), operation time (*p* = 0.09), postoperative infection rate (*p* = 1.00), site of primary tumor (*p* = 0.15), or level of operated spinal involvement (*p* = 0.79). Group A had a shorter postoperative hospital stay in both acute (in orthopedic or neurosurgical ward) (*p* = 0.0047) and chronic stages (total length of hospitalization, including referral to other departments) (*p* = 0.0032); in addition, it had a better preoperative revised Tokuhashi score (*p* < 0.0001, Table 4).

**Table 4:** Comparison of patient characteristics in Groups A and B.

	Group A (n = 37)		Group B (n = 52)		*p* value
Sex, male/female	22/15		32/20		1.00
	**Mean**	**95% CI**	**Mean**	**95% CI**	
Age	57.8	53.7–61.9	56.2	52.9–59.4	0.54
	**Median**	**95% CI**	**Median**	**95% CI**	
Acute hospital stay	11	9–12	14.5	12–18	0.0047**
Chronic hospital stay	12	10–15	23	16–28	0.0032**
Tomita score	6	4.1–6	6	6–7	0.0992
Revised Tokuhashi score	9	8–10	7	6–8	<0.0001**
Blood loss	550	300–900	825	427–1126	0.19
Operation time	162	156–211	214.5	186–233	0.09
	**Number of patients**	**Percentage (%)**	**Number of patients**	**Percentage (%)**	
Postoperative infection (%)	6	16.2	8	15.4	1.00
Primary tumor					0.15
Rapid growth	18	48.6	28	53.8	
Moderate growth	9	24.3	18	34.6	
Slow growth	10	27.0	6	11.5	
Level of operated spine					0.79
Cervical	1	2.7	2	3.8	
Upper thoracic (T1–T6)	8	21.6	24	46.2	
Lower thoracic (T7–T12)	16	43.2	23	44.2	
Lumbar and sacrum	12	32.4	3	5.8	

CI, confidence interval.

**p < 0.001.

There were no statistically significant differences between Groups B1 and B2 in age (*p* = 0.92), sex (*p* = 0.08), acute stage hospital stay (*p* = 0.63), chronic stage hospital stay (*p* = 0.29), Tomita score (*p* = 0.43), revised Tokuhashi score (*p* = 0.07), blood loss (*p* = 0.47), operation time (*p* = 0.24), postoperative infection rate (*p* = 0.42), site of primary tumor (*p* = 0.21), or level of operated spinal involvement (*p* = 0.70, Table 5).

**Table 5:** Comparison of patient characteristics in Groups B1 and B2.

	Group B1 (n = 18)		Group B2 (n = 34)		*p* value
Sex, male/female	8/10		24/10		0.08
	**Mean**	**95% CI**	**Mean**	**95% CI**	
Age	56.4	49.7–63.1	56.1	52.3–59.8	0.92
	**Median**	**95% CI**	**Median**	**95% CI**	
Acute hospital stay	18.5	9.4–29.6	14	12–16.3	0.63
Chronic hospital stay	29	12.8–44.8	20.5	15–27.2	0.29
Tomita score	6	5–6.6	6.5	6–8	0.43
Revised Tokuhashi score	6	4–7	7.5	6–8	0.07
Blood loss	1000	364–1398	600	300–1224	0.47
Operation time	232.5	200–256	192	172–230	0.24
	**Number of patients**	**Percentage (%)**	**Number of patients**	**Percentage (%)**	
Postoperative infection (%)	4	22.2	4	11.8	0.42
Primary tumor					0.21
Rapid growth	11	61.1	17	50.0	
Moderate growth	7	38.9	11	32.4	
Slow growth	0	0	6	17.6	
Level of operated spine					0.70
Cervical	1	5.6	1	2.9	
Upper thoracic (T1–T6)	11	61.1	16	47.1	
Lower thoracic (T7–T12)	6	33.3	14	41.2	
Lumbar and sacrum	0	0	3	8.8	

CI, confidence interval.

### Survival analysis

**Fig 2:**
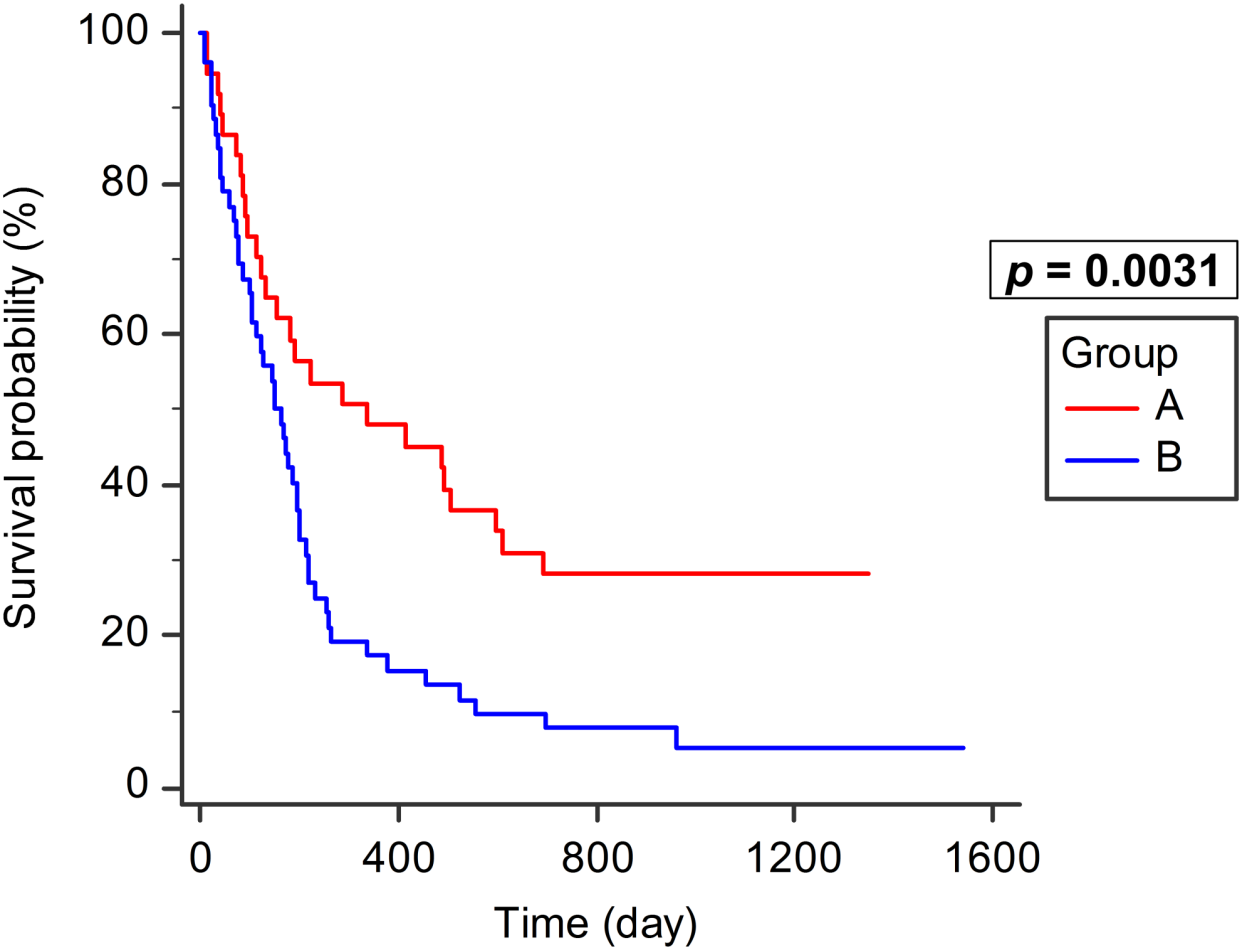
Survival in Groups A and B. Observed survival was better in Group A (intact motor function, n = 37) than in Group B (motor deficit, n = 52, *p* = 0.0031).

The median for postoperative overall survival was 182 days with 95% confidence interval (CI) of 132–219 days. The estimated survival rates at 3, 6, and 12 months were 70.8%, 49.3%, and 28.7%, respectively. The median survival was 338 days in Group A (95% CI: 132–599) and 150 days in Group B (95% CI: 105–198). The observed survival was better in Group A than in Group B (*p* = 0.0031; Fig 2), but was not significantly different between Groups B1 and B2 (*p* = 0.52).

**Fig 3:**
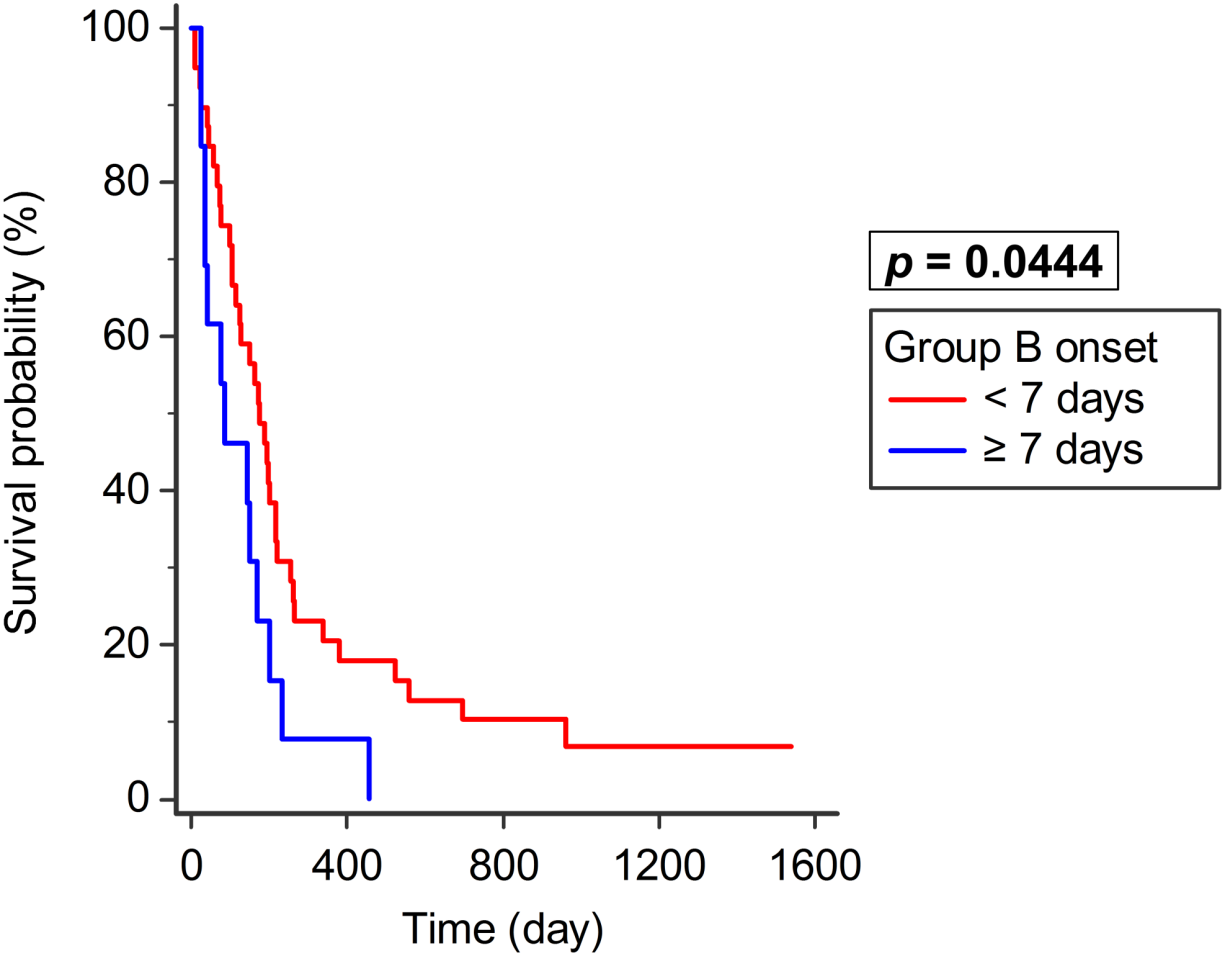
Group B survival—Timing of surgery. In Group B (motor deficit), survival was better in patients who underwent surgery within 7 days (n = 39) than in those who did after 7 days (n = 13, *p* = 0.0444).

In Group B (n = 52), the median survival was 176 days for patients who underwent surgery within 7 days (n = 39, 95% CI: 115–217) and 87 days for those who underwent surgery after more than 7 days (n = 13, 95% CI: 36–168). Survival rate was thus better in patients who underwent surgery within 7 days than in those who underwent surgery after 7 days (*p* = 0.0444; Fig 3). There were no significant differences in Tomita score and revised Tokuhashi score between the two subgroups in Group B (early operation group: before 7 days; delayed operation group: after 7 days). The *p* values in Tomita score and revised Tokuhashi score were 0.1332 and 0.1848 respectively.

**Fig 4:**
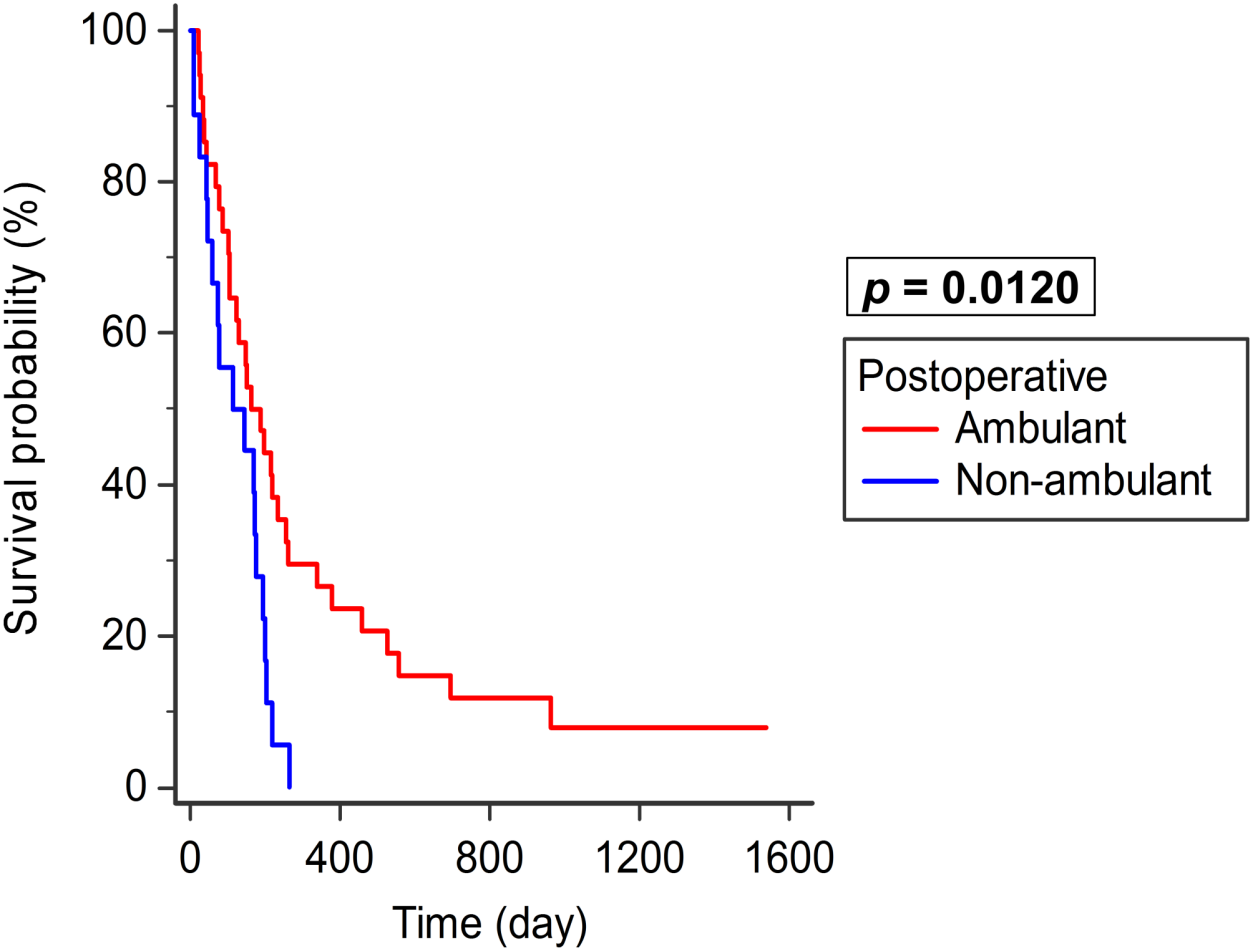
Group B survival—Postoperative ambulation. Better survival was observed in postoperative ambulant patients (n = 34) than in nonambulant patients (n = 18) in Group B (motor deficit, *p* = 0.0120).

In Group B, patients who were postoperatively ambulant (Frankel grade D and E) had a median survival of 164 days (n = 34, 95% CI: 105–256), whereas those who were nonambulant (Frankel grade A–C) had a median survival of 115 days (n = 18, 95% CI: 58–176). Better survival was observed in postoperative ambulant patients than in nonambulant patients in Group B (*p* = 0.0120; Fig 4).

### Neurological outcome

**Fig 5:**
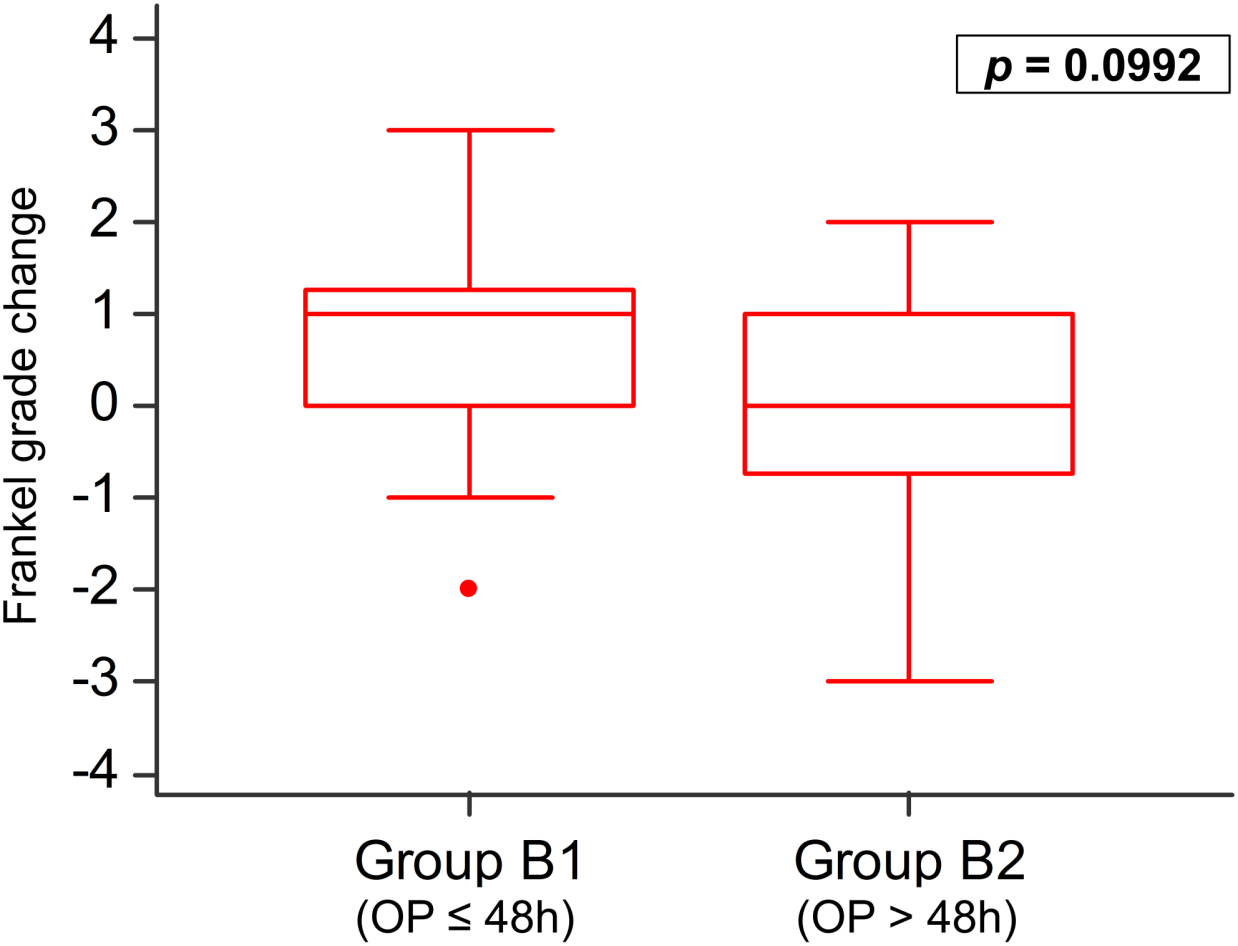
Group B neurological recovery—Timing of surgery. Group B (motor deficit) was subdivided into the group that underwent surgery within 48 h (Group B1, n = 18) and those who did after 48 h (Group B2, n = 34). Comparing both groups, there were no statistically significant differences in Frankel grade change (*p* = 0.0992).

In Group B, earlier surgery within 48 h (Group B1, n = 18) led to an average improvement by one Frankel grade. In patients whose surgery was delayed for more than 48 h, there was no improvement in Frankel grade. There were no statistically significant differences in Frankel grade improvement (*p* = 0.0992; Fig 5) between the groups. Comparing pre and postoperative Frankel grade by Wilcoxon signed-rank test in Group B1 yielded an improvement tendency (*p* = 0.0674; S1 Fig), but it was not observed in Group B2 (*p* = 0.9723; S2 Fig). Surgery within 48 h seemed to improve the neurological outcome.

### Factors influencing survival

Among the 89 MSCC patients treated with palliative decompression, lower Tomita score (≤5 points), higher revised Tokuhashi score (≥9 points), better preoperative Frankel grade, and younger age (≤55 years) all led to better survival.

**Fig 6:**
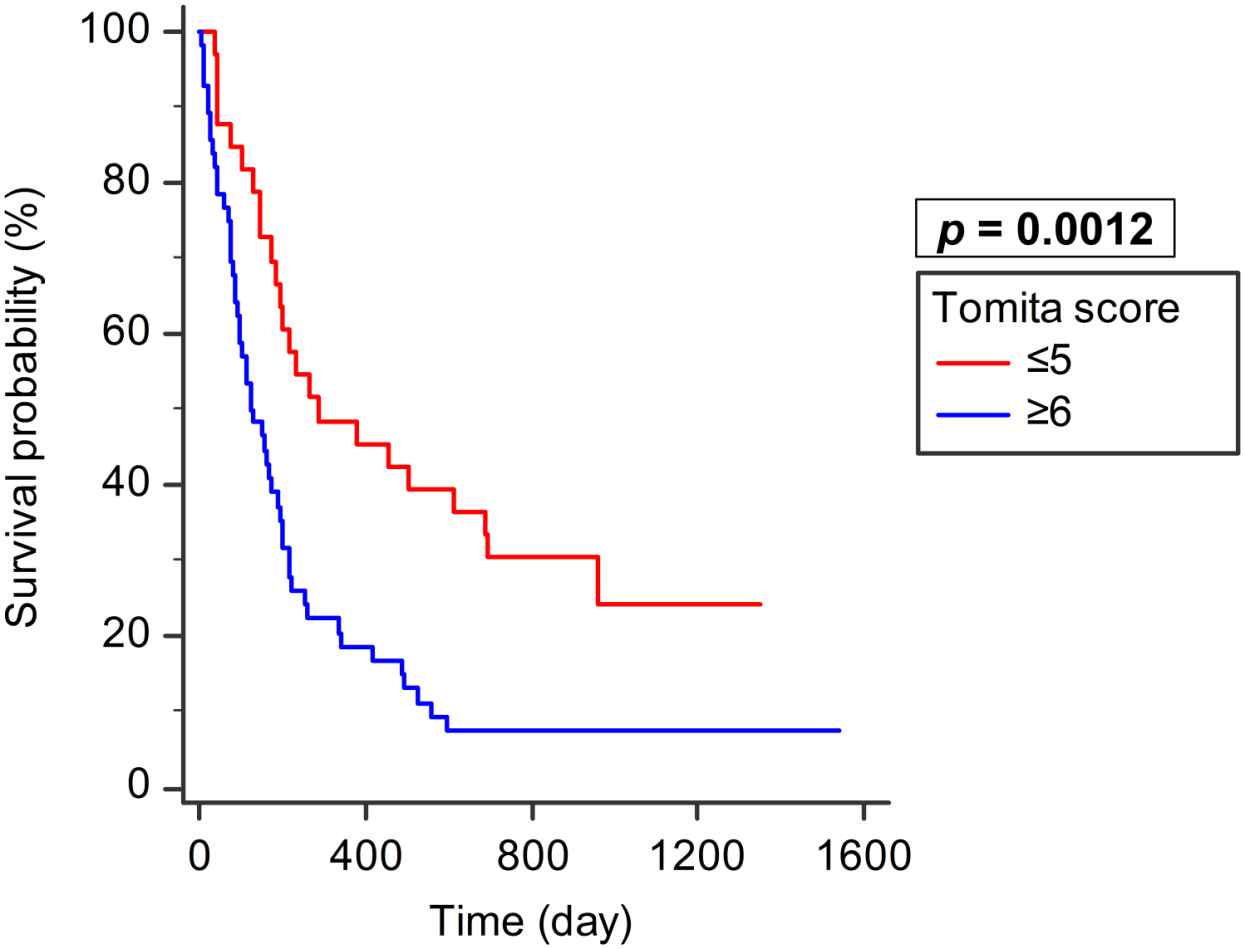
Tomita score and survival. Patients with a Tomita score of ≤5 points (n = 33) had significantly better survival than those with ≥6 points (n = 56, *p* = 0.0012).

**Fig 7:**
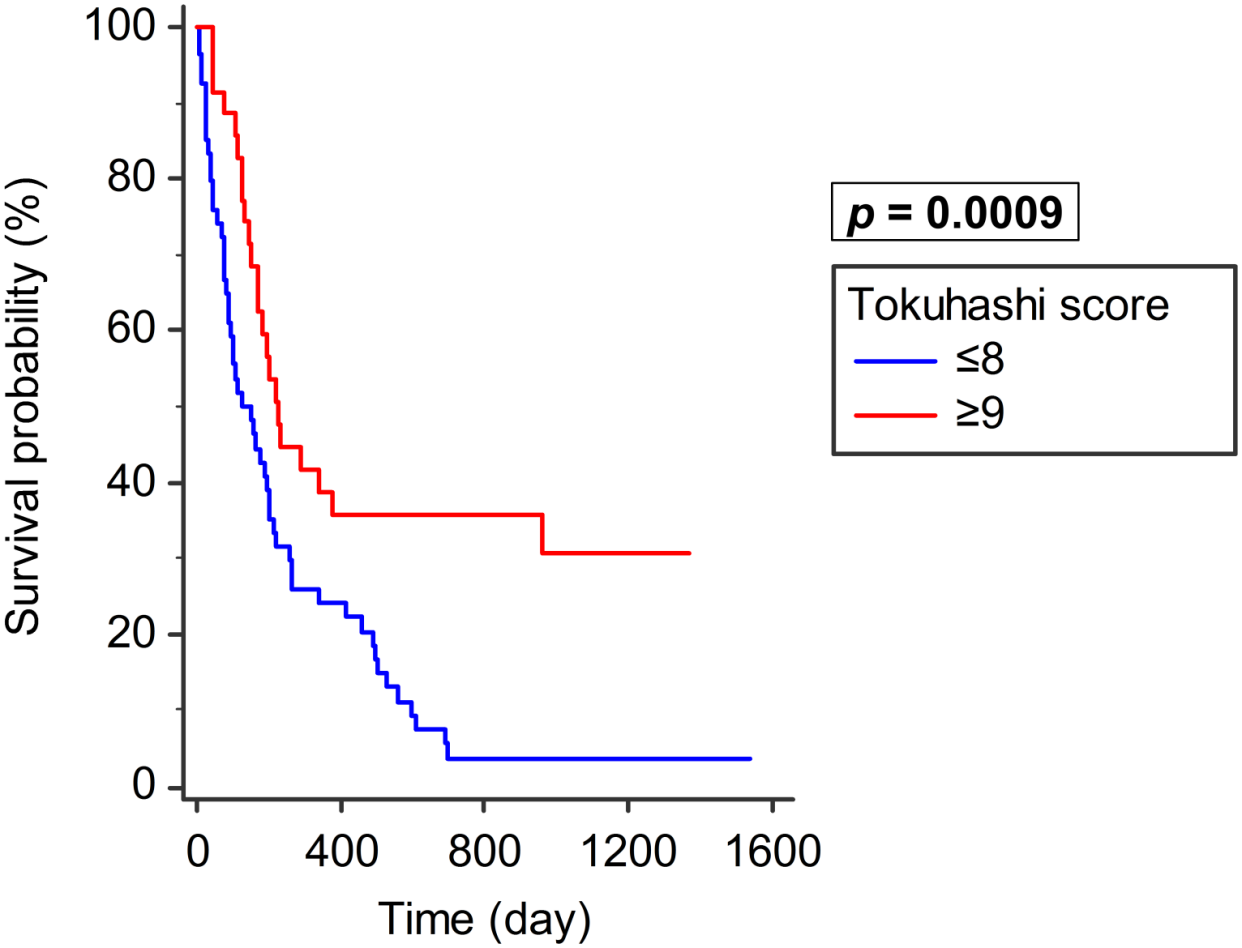
Revised Tokuhashi score and survival. Patients with revised Tokuhashi score of ≥9 points (n = 35) had significantly better survival than those with ≤8 points (n = 54, *p* = 0.0009).

Patients with a Tomita score of ≤5 points (n = 33, median survival: 286 days, 95% CI: 193–691) had a significantly better survival than those with ≥6 points (n = 56, median survival: 124 days, 95% CI: 92–188; *p* = 0.0012; Fig 6). Patients with a revised Tokuhashi score of ≥9 points (n = 35, median survival: 225 days, 95% CI: 168–962) had significantly better survival than those with ≤8 points (n = 54, median survival: 149 days, 95% CI: 87–198; *p* = 0.0009, Fig 7).

**Fig 8:**
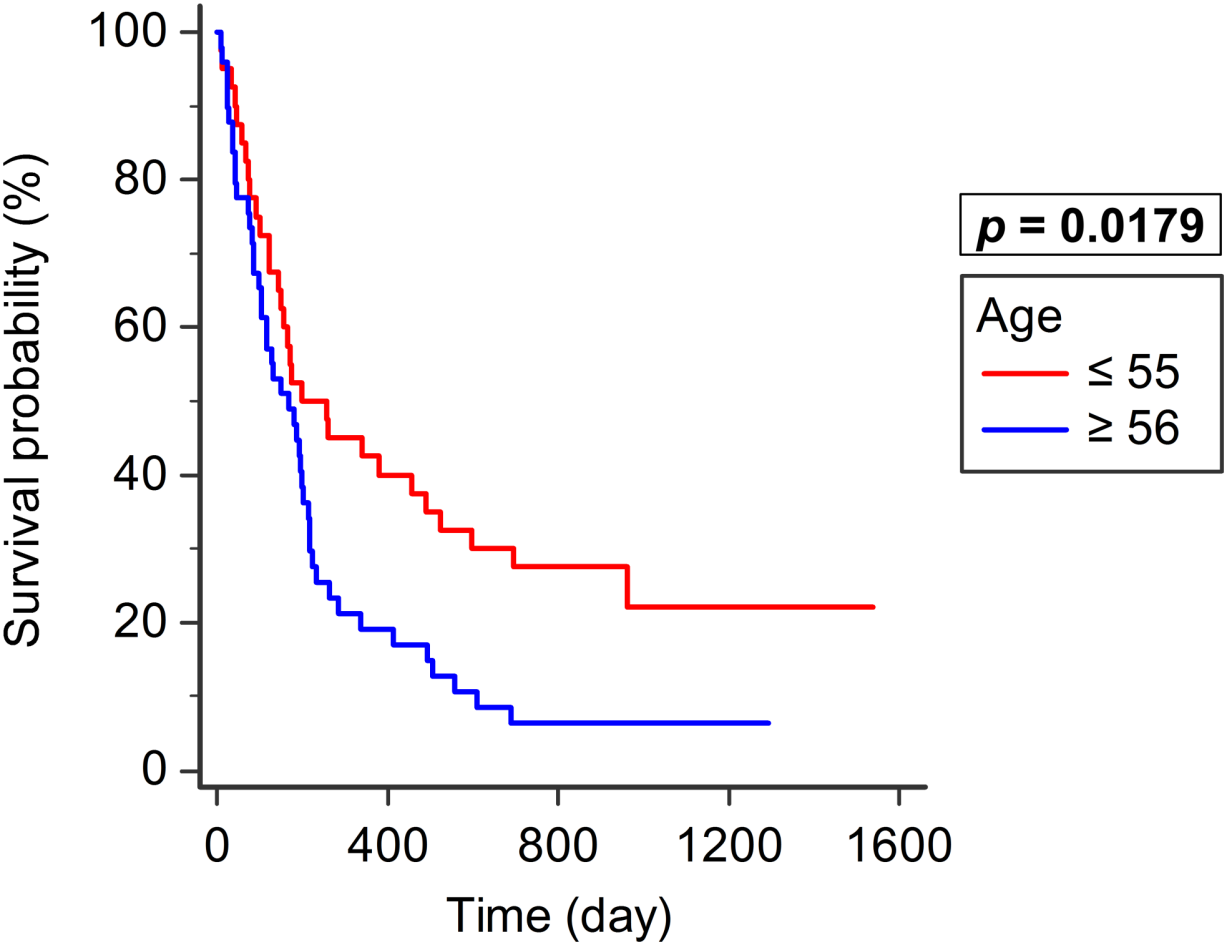
Age and survival. Patients aged ≤55 years (n = 40) had significantly better survival than those aged ≥56 years (n = 49; *p* = 0.0179).

Patients were divided into five groups, according to preoperative Frankel grade (A–E). Survival was significantly different between the five groups (*p* < 0.0001). Better survival was observed in patients with grades closer to E. In addition, patients aged ≤55 years (n = 40, median survival: 200 days, 95% CI: 149–490) had significantly better survival than those aged ≥56 years (n = 49, median survival: 168 days, 95% CI: 104–202; *p* = 0.0179; Fig 8).

## Discussion

This study analyzed 89 patients with MSCC who received palliative decompression. Comparing the patients who underwent palliative decompression with intact motor function (Group A) and motor deficit (Group B), the former had a significantly better survival and higher revised Tokuhashi score (*p* = 0.0031 and <0.0001, respectively). Palliative decompression led to better survival in MSCC patients before the onset of motor deficit. Patients had better revised Tokuhashi score at that time and it was related to better survival [20, 21]. In our study, the difference in survival by revised Tokuhashi score between Groups A and B was significant (*p* < 0.0001). The revised Tokuhashi score is composed of 6 categories. We found that the difference in survival outcome between Groups A and B was most dependent upon the performance status and degree of palsy, which deeply influence the revised Tokuhashi score. In other categories of revised Tokuhashi score and Tomita score, no significant differences were found between Groups A and B. It implies that operation before palsy (Group A) leads to a better survival. In previous studies, with better Tomita or revised Tokuhashi score, more aggressive intervention is suggested, such as en bloc, wide, or marginal excision [2, 3]. Our study demonstrates that palliative decompression achieves acceptable outcome in patients with good Tomita or revised Tokuhashi score.

In Group B, surgery within 48 h led to Frankel grade improvement, albeit not statistically significant (*p* = 0.0992). Surgery delayed for less than 7 days had better survival (*p* = 0.0444) than those delayed for more than 7 days. There were no significant differences in Tomita and revised Tokuhashi scores between the two subgroups in Group B. It implied that better survival was not related to better scores. Emergency surgery within 48 h tended to improve neurological outcome, whereas delaying up to 7 days still achieved a better survival than delaying it for more than 7 days. These findings are compatible with the literature that emergency surgery leads to better neurological recovery [7, 22, 23]. However, limited studies have shown that emergency surgery results in better survival. Park et al. analyzed MSCC patients from non-small cell lung cancer (NSCLC) and found better survival was related to surgery within 72 h [17]. The difference may be due to different patient groups (NSCLC vs. multiple types of tumor) and different surgical techniques. We focused on palliative decompression and excluded other types of surgery, such as vertebral column resection. To our knowledge, this is the first study showing that preoperative motor deficit MSCC patients have a better survival, despite the delay of palliative decompression up to 7 days.

Several studies demonstrate that survival is better in preoperative ambulant patients [13, 14, 15, 16]. Preoperative ambulatory status has a positive effect on postoperative ambulatory outcome [13, 20, 24, 25, 26]. However, few data are available to prove that survival is better in postoperative ambulant patients [11, 17]. One report showed that the functional outcomes did not directly influence patient survival [27]. This study focused only on thoracic spine, and multiple types of surgical techniques were enrolled, leading to different results regarding postoperative ambulation and survival. Our study exhibits that under palliative decompression, postoperative ambulant patients have better survival.

Tomita score and revised Tokuhashi score are widely applied in predicting survival in MSCC patients [20, 21, 28, 29]. In a prospective study by Morgen et al., the author classified both Tomita score and revised Tokuhashi score into three groups and found significantly different survival between the three groups [21]. Our study classified patients into only two groups and achieved significance at the cut-off point of 5 for the Tomita score, and 9 for the revised Tokuhashi score.

In a previous study, patients with a Tomita score of ≤5 points were suggested to receive intralesional, marginal, or wide excision. [10] Palliative decompression that was originally considered for patients with a Tomita score of ≥6 points was applied to those patients (≤5 points), which still resulted in a better survival. In patients with a revised Tokuhashi score of ≥9 points, the predicted survival was more than 6 months. Excisional surgery was considered in case of a single lesion that did not metastasize to major internal organs. [19] We performed palliative decompression in those patients and still achieved a better survival.

For the present study showed for the first time that among MSCC patients treated with palliative decompression, those ≤55 years have a significantly better survival than those ≥56 years. It implies that younger patients may benefit more from palliative decompression.

Overall, patients can benefit from an earlier operation when diagnosed with MSCC in different stages, irrespective of the occurrence of motor deficits. An operation performed before the occurrence of motor deficits can lead to a better survival. After the onset of motor deficits, an early operation performed within 48 h could improve neurological recovery. Delaying surgery within 7 days still improves the survival, although at that time point (2–7 days), neurological recovery could not be significantly improved.

There are three major limitations in the present study. First, it was a retrospective nonrandomized study, and the decisions on surgery were not determined using the same criteria. Second, detailed chemotherapy and radiotherapy options were not reviewed. However, the patients in our study had up to 14 types of different tumor origins, and discussing chemotherapy or radiotherapy between different tumor types was hard to draw a conclusion. Third, the sample size was relatively small (n = 89). In the future, a multicenter, prospective, randomized trial enrolling a large number of patients will be of high value to verify the key factors influencing survival in MSCC patients.

## Conclusion

We can improve the survival of MSCC patients with palliative decompression before motor deficit onset. After the occurrence of motor deficits, the survival can still be improved with early surgery within 7 days of the onset. Overall survival was better in patients aged ≤55 years.

## Supporting information

S1 Fig Paired pre and postoperative Frankel grade in Group B1. Comparing pre and postoperative Frankel grade by Wilcoxon signed-rank test in Group B1 yielded an improvement tendency (*p* = 0.0674). The Y-axis is Frankel grade (1 = grade A, 2 = grade B, 3 = grade C, 4 = grade D, 5 = grade E).
(TIF)

S2 Fig Paired pre and postoperative Frankel grade in Group B2. There was no significant difference between pre and postoperative Frankel grade in Group B2 (*p* = 0.9723). The Y-axis is Frankel grade (1 = grade A, 2 = grade B, 3 = grade C, 4 = grade D, 5 = grade E).
(TIF)
