# Supplementary figures and images for: Metastatic spinal cord compression (MSCC) treated with palliative decompression: Surgical timing and survival rate
Source: PLoS One. 2017 Dec 29;12(12):e0190342. doi: 10.1371/journal.pone.0190342 (PMC5747484; doi:10.1371/journal.pone.0190342)

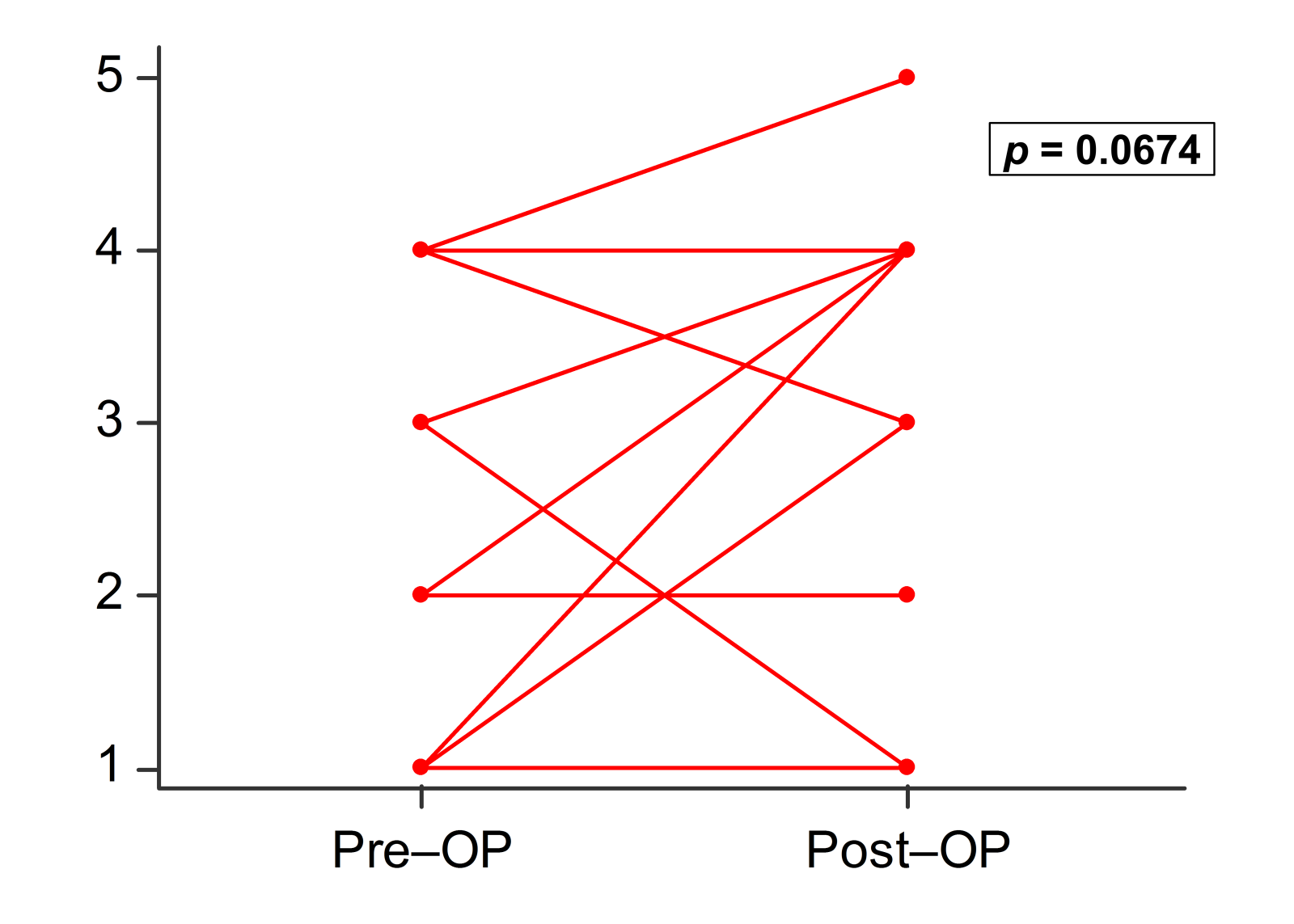

Supplement: S1 Fig — Comparing pre and postoperative Frankel grade by Wilcoxon signed-rank test in Group B1 yielded an improvement tendency (p = 0.0674). The Y-axis is Frankel grade (1 = grade A, 2 = grade B, 3 = grade C, 4 = grade D, 5 = grade E). (TIF) [file pone.0190342.s001.tif]

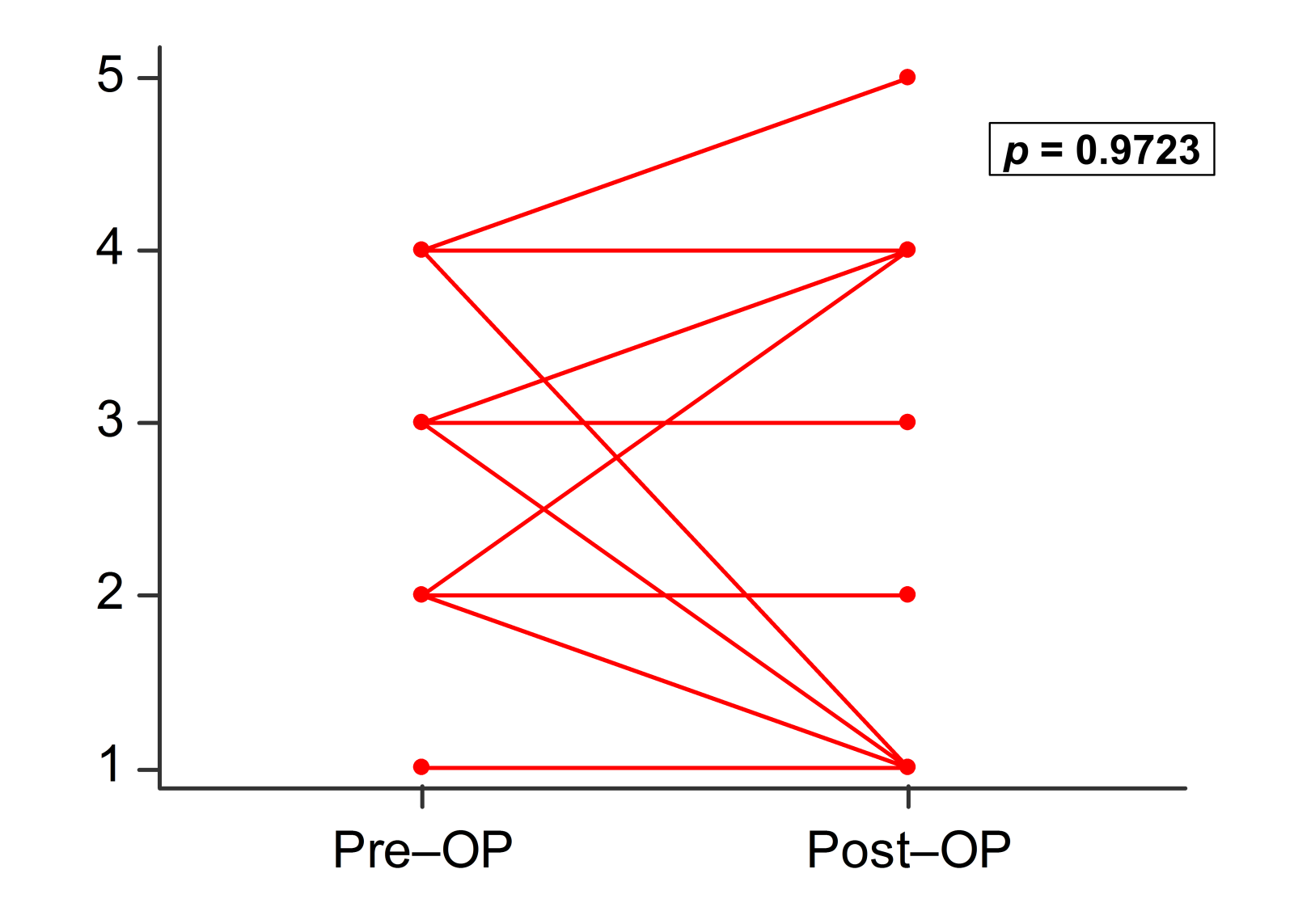

Supplement: S2 Fig — There was no significant difference between pre and postoperative Frankel grade in Group B2 (p = 0.9723). The Y-axis is Frankel grade (1 = grade A, 2 = grade B, 3 = grade C, 4 = grade D, 5 = grade E). (TIF) [file pone.0190342.s002.tif]
